# Update of ALDH as a Potential Biomarker and Therapeutic Target for AML

**DOI:** 10.1155/2018/9192104

**Published:** 2018-01-03

**Authors:** Xiangchou Yang, Rongxin Yao, Hong Wang

**Affiliations:** ^1^Department of Hematology, The Second Affiliated Hospital and Yuying Children's Hospital of Wenzhou Medical University, Wenzhou, China; ^2^Department of Rheumatology, The Second Affiliated Hospital and Yuying Children's Hospital of Wenzhou Medical University, Wenzhou, China

## Abstract

Studies employing mouse transplantation have illustrated the role of aldehyde dehydrogenase (ALDH) defining hematopoietic stem cells (HSCs) and leukemia stem cells (LSCs). Besides being a molecular marker, ALDH mediates drug resistance in AML, which induces poor prognosis of the patients. In AML patients, either CD34^+^ALDH^br^ population or CD34^+^CD38^−^ALDH^int^ population was found to denote LSCs and minimal residual disease (MRD). A bunch of reagents targeting ALDH directly or indirectly have been evaluated. ATRA, disulfiram, and dimethyl ampal thiolester (DIMATE) are all shown to be potential candidates to open new perspective for AML treatment. However, inconsistent results have been shown for markers of LSCs, which makes it even more difficult to differentiate LSCs and HSCs. In this review, we elevated the role of ALDH to be a potential marker to define and distinguish HSCs and LSCs and its importance in prognosis and target therapy in AML patients. In addition to immunophenotypical markers, ALDH is also functionally active in defining and distinguishing HSCs and LSCs and offers intracellular protections against cytotoxic drugs. Targeting ALDH may be a potential strategy to improve AML treatment. Additional studies concerning specific targeting ALDH and mechanisms of its roles in LSCs are warranted.

## 1. Introduction

Acute myeloid leukemia (AML) is a clonal disorder defined by the accumulation of abnormally differentiated myeloid cells, which lead to a series of fetal clinical problems. They are heterogeneous in morphologic and cytogenetic features, and their prognoses are extremely different and individualized.

Current treatment of AML, either cytotoxicity or bone marrow transplantation, mainly targets bulk leukemia cells, with some variations depending on the types of AML, cytogenetic analysis, and the patient's personal conditions. Standard treatment, although improving the response of some specific types of AML, has not changed the outcome of most patients dramatically. A variety of new trials, including targeted immune therapy, have been developed and tested in clinical practice, but each of these concepts has its particular merits and inherent problems, and none of them have changed the outcome of AML significantly [1]. Thus, exploring new diagnostic, prognostic, and therapeutic tools for AML is necessary. Mounting evidences showed that AML arises from genetic changes in HSCs or hematopoietic progenitors [2, 3] and is organized as a hierarchy that is maintained by LSCs, which in turn initiates the abnormal differentitation program and leads to the production of terminal blast cells [4, 5]. Understanding the biology of these cells and how they transform to AML is one key to new treatment alternatives.

Many studies have been initiated to characterize LSCs and the difference between LSC and normal HSC, which is critical to understand the leukemogenic process and facilitate new therapy strategy targeting LSC while sparing normal HSC counterpart. Like HSC, LSCs are enriched in the CD34^+^CD38^−^ fraction of the AML cells [6], with more immunophenotypical markers, such as CD123, CD45RA, or CD93 [6–8]. However, inconsistent results have been shown for markers of LSCs, which makes it even more difficult to differentiate LSCs from HSCs [6, 9]. Therefore, immunophenotypical surface markers are not reliable to define LSCs. ALDH is proved to be an interesting candidate both in normal HSCs and in leukemia transformation.

## 2. ALDH Is a Potential Marker for HSCs in Both Human and Mouse Bone Marrow

The aldehyde dehydrogenase (ALDH) is a group of intracellular enzymes that participate in cellular detoxification, differentiation, and drug resistance by oxidation of cellular aldehydes [10]. To date, 19 ALDH genes are identified within the human genome. These genes share sequence homology and overlapping activities including protein chaperone activity, metabolism of retinoids, reactive oxygen species (ROS), and reactive aldehydes.

Sequencing of the human genome and subsequent identification of mutations in ALDH genes associated with loss of ALDH enzyme activity have led to the identification of biological process of various normal and cancer stem cells [11–13]. Of all the ALDHs, ALDH1A1 was found to be most highly expressed in both murine and human HSCs and immature progenitors [14, 15]. CD34^+^ALDH^bright^ (ALDH^br^) cells from human umbilical cord blood (UCB) contain all the NOD/SCID engrafting cells in the CD34^+^ cells and HSC assessment by ALDH activity yields highest correlation with conventional analytic methods [16]. In mobilized peripheral blood stem cells (PBSC), the side-scatter low ALDH^br^ (SSC^lo^ALDH^br^) cells are highly enriched for human HSC. Furthermore, ALDH are positively correlated with the capacity of PBSC graft during HSC transplantation (HSCT) [17], ALDH^br^ cells correlated best to both platelet and neutrophil reconstitution [18].

Although ALDH1a1 knockout in mice showed no fetal defects of HSCs [19], deletion of both ALDH1a1 and ALDH3a1 could severely block B-cell development and reduce the number of HSCs [20]. Diethylaminobenzaldehyde (DEAB, an inhibitor of ALDH) or siRNA of ALDH could impede the differentiation of murine HSCs but induce amplification of short-term HSCs (ST-HSC). Inhibition of ALDH, primarily ALDH1a1, with all-transretinoic acid (ATRA), could block DEAB-mediated expansion of ST-HSC, indicating that ALDH1a1 is involved in regulating HSC differentiation via increasing retinoid signaling [21]. The levels of ALDH activity in adult murine HSCs, however, are quite heterogeneous. ALDH^(br)^ population does not seem to contain known HSCs or progenitors, while the ALDH^(dim)^ together with CD48^−^EPCR^+^ cells yields high levels of engraftment and ALDH^(int)^ cells can give long-term HSCs (LT-HSCs) [22].

The molecular basis for ALDH function in HSCs has not been extensively studied. There was evidence showing that ALDH is involved in metabolism of ROS and reactive aldehydes in HSCs [19, 23], both of which may play important roles in HSC biology and leukemia transformation. Another mechanism behind ALDH functions is through retinoic acid (RA) signaling, as ALDH1 catalyzes the oxidation of RA, which can drive the transcription of target genes via a heterodimer formed by RA receptors (RAR) and retinoid-X-receptor [24]. RA signaling pathway is important in regulating HSC self-renewal [25]. Previous study showed that inhibition of ALDH1 induced an decrease of RA activity, together with decrease of HSC differentiation in cord blood [21, 26], indicating the ALDH mediates its effects on HSC fate via its contribution on retinoid production and RA signaling pathway. Besides ALDH/RAR axis, HOXB4 and Notch signaling were also shown to be involved in regulating HSC self-renewal independent of RA [26].

Taken together, these findings demonstrate that ALDH, primarily ALDH1, plays critical roles in the biology of HSCs and is an important marker for HSCs.

## 3. ALDH Might Be Involved in Leukemia Development and Is a Potential Marker for LSCs

In addition to their roles in normal HSCs and hematopoiesis, ALDH may be involved in leukemia transformation. Knockout of ALDH2 in a murine model of Fanconi anemia (FA), Aldh2^(−/−)^Fancd2^(−/−)^ mice spontaneously develop acute leukemia [27]. As the target of the oncogenic homeoprotein TLX1/HOX11, ALDH1A1 regulated by TLX1 can profoundly perturb murine hematopoiesis by promoting myeloid differentiation at the expense of lymphopoiesis [28]. With both ALDH1A1 and ALDH3A1 deletion, NUP98-HOXA10 homeodomain fusion protein (NA10HD) can promote the development of leukemia with B220^+^ and varied levels of CD11b, though NA10HD alone only induces a rapid and marked expansion of HSC in vitro without malignant transformation [20, 29], strongly indicating the important role of ALDH in leukemia initiation.

In addition to possible involvement in leukemogenesis, more and more studies have been done to investigate the role of ALDH in LSCs. Early data demonstrated that ALDH^+^ cells, largely overlapping with CD34^+^ cells, are enriched for LSCs. However, a significant amount of ALDH^br^ cells with LSC characteristics did not express the CD34+CD38^–/low^ phenotype, indicating immunophenotype alone is not adequate to mark LSCs [29, 30]. Further dissecting ALDH^br^ population showed that side-scatter low (SSC^lo^)ALDH^br^ population might define primitive LSC and confer an inferior prognosis in AML patients [31, 32]. Gerber et al., however, identified an additional ALDH^int^ population in CD34^+^CD38^−^ cells which are capable of leukemia initiation in NSG mice and believed to be the marker of LSCs as well as minimal residual disease (MRD) [33]. The inconsistent findings from different studies could be due to the different origins of AMLs, and AML cases with CD34^+^CD38^−^ALDH^br^ leukemia cells might be derived from more primitive cells, possibly translating to a poorer prognosis.

## 4. ALDH Helps Distinguish LSCs from Normal HSC 

Mounting evidences indicate that leukemia cells in patients with AML are derived from LSC. Residual, physiological HSCs exist alongside LSC, with heterogeneous dominance of LSC over HSC in individual patients. One of the challenges in AML treatment may be largely due to the difficulty to distinguish LSCs from their normal HSC counterpart. In recent years, much effort has focused on ways to specifically distinguish and eliminate LSCs.

Although ALDH denotes both HSCs and LSCs, its activity in these cells is shown to be different, with higher ALDH in normal HSCs than in LSCs [34]. Studies by Ran et al. defined LSCs with CD34^+^ALDH^br^ [31, 32]. However, in ALDH^br^ population, normal HSCs were found, though with very rare proportion [32], indicating ALDH^br^ is not adequate to distinguish LSCs from HSCs in some patients. Besides, in some patients, ALDH^br^ population, although exhibited stem cell characteristics, does not contain the leukemia-specific cytogenetic abnormalities, indicating that they might be normal HSC compartment. Further evidence showed the LSCs fell in ALDH^int^ population which was absent in normal HSCs and bone marrow cells with remission [35]. Containing leukemia-specific cytogenetic abnormalities, this population is more closely related to their original LSCs.

Based on all these findings, ALDH activity varies in AML and also differs from patient to patient. The difference in spectrum and relevance of ALDH activity in the putative LSC populations demonstrates that, in addition to phenotypic and genetic, the leukemia cells are heterogeneous functionally. By acknowledging these differences, we can better understand leukemia development and prognosis, which in turn can facilitate the efforts to find new therapeutic targets for this disease.

## 5. ALDH as a Potential Target in the Treatment of AML

In addition to its role as an important marker for LSCs and MRD, measuring and targeting ALDH may have a variety of practical benefits in the clinical management of AML. One of the challenges of ALDH in AML treatment is that it mediates resistance to widely used chemotherapeutic reagents. ALDH was found to meditate the irreversible detoxification of prodrug cyclophosphamide (a substrate of ALDH). For some AMLs with high ALDH activity, this might contribute to chemotherapy resistance and treatment failure [36, 37]. Additional evidence showed induced ALDH1 expression by cytokines in the bone marrow cells resulted in increased resistance to 4-hydroperoxycyclophosphamide (4-HC) which was effective in purging bone marrow or peripheral blood cell collections before autologous transplantation [38, 39], while reducing the expression of ALDH could sensitize the leukemia cells to 4-HT [40]. The functional activity of ALDH, together with the differential expression of ALDH in LSCs and HSCs, can provide a potential therapeutic window to target LSCs specifically.

DEAB, an ALDH-specific inhibitor, has been shown to deregulate human HSC self-renewal by interfering with endogenous RA biosynthesis [26], while ATRA may act directly on murine HSC to enhance their maintenance in culture [41]. RA's function in AML is not clear yet. Some study showed ATRA could induce the differentiation of ALDH^int^ LSCs in nonacute promyelocytic leukemia (APL) [42], indicating ATRA could be a promising candidate targeting LSCs in AML.

More effort has been taken to search for better reagents targeting specifically the ALDH denoted LSCs. Disulfiram (DSF), as an inhibitor of ALDH, could selectively eradicate AML LSCs by simultaneous induction of ROS-JNK and inhibition of NF-kB and Nrf2 [43, 44]. Lately, in Down syndrome-associated AML, DSF with copper was found to overcome bortezomib and cytarabine resistance in ALDH^br^ LSCs via inducing apoptosis and proteasome inhibition in the leukemia cells [45], implying DSF could offer a new treatment option for the AML patients.

Another well-characterized ALDH inhibitor dimethyl ampal thiolester (DIMATE) was assessed on LSCs compared with HSCs. Intriguingly, DIMATE is highly active against LSCs, but, unlike conventional chemotherapy, it is not toxic for healthy HSCs [26, 46]. Therefore, DIMATE presents to be a very promising drug specifically targeting LSCs while sparing normal HSCs, opening a new therapeutic perspective in AML.

Furthermore, with high-throughput screening (HTS), over 64,000 compounds were examined to discover novel and selective inhibitors of ALDH1A1, and more than 30 hits were found and were further analyzed [47, 48]. Although the function of those compounds is unknown in AML, this represents a starting point for the development of highly potent and selective inhibitors of ALDH and may be utilized in AML treatment.

Besides exploring new candidate reagents, conventional therapies can also be improved with better understanding of ALDH. Collecting enough HSCs for BMT has always been challenging in clinical practice, and ex vivo expansion of the cells attracts wide attention of many researchers worldwide. ALDH, as a functionally active marker in HSCs, inhibition might be a means to amplify ST-HSC for transplantation purpose, based on the findings that ATRA could block DEAB-mediated expansion of ST-HSC in culture [21].

## 6. Conclusions

In summary, the ALDH family, together with its substrates, may play important roles in normal HSCs and leukemia transformation, prognosis, and treatment in AML. Understanding the biology of ALDH and the difference of its roles in HSCs and LSCs is critical to develop new therapeutic strategies selectively targeting LSCs while sparing normal HSCs. Inhibitors of ALDH are potential candidates for improving AML treatment, especially for relapsed/refractory patients, as their safety is well described in HSC population [26, 42]. However, despite all the progress, further evaluation of the roles of this gene family in hematopoietic malignancies and the heterogeneity in LSCs is warranted.

## References

[1] Lichtenegger F. S., Krupka C., Haubner S. (2017). Recent developments in immunotherapy of acute myeloid leukemia. *Journal of hematology and oncology*.

[2] Ugale A., Norddahl G. L., Wahlestedt M. (2014). Hematopoietic stem cells are intrinsically protected against MLL-ENL-mediated transformation. *Cell Reports*.

[3] Sykes S. M., Kokkaliaris K. D., Milsom M. D., Levine R. L., Majeti R. (2015). Clonal evolution of preleukemic hematopoietic stem cells in acute myeloid leukemia. *Experimental Hematology*.

[4] Hanekamp D., Cloos J., Schuurhuis G. J. (2017). Leukemic stem cells: identification and clinical application. *International Journal of Hematology*.

[5] Hope K. J., Jin L., Dick J. E. (2004). Acute myeloid leukemia originates from a hierarchy of leukemic stem cell classes that differ in self-renewal capacity. *Nature Immunology*.

[6] Al-Mawali A., Gillis D., Lewis I. (2016). Immunoprofiling of leukemic stem cells CD34+/CD38-/CD123+ delineate FLT3/ITD-positive clones. *Journal of Hematology & Oncology*.

[7] Iwasaki M., Liedtke M., Gentles A. J., Cleary M. L. (2015). CD93 marks a non-quiescent human leukemia stem cell population and is required for development of MLL-rearranged acute myeloid leukemia. *Cell Stem Cell*.

[8] Kersten B., Valkering M., Wouters R. (2016). CD45RA, a specific marker for leukaemia stem cell sub-populations in acute myeloid leukaemia. *British Journal of Haematology*.

[9] Quek L., Otto G. W., Garnett C. (2016). Genetically distinct leukemic stem cells in human CD34-acute myeloid leukemia are arrested at a hemopoietic precursor-like stage. *The Journal of Experimental Medicine*.

[10] Yang C. K., Wang X. K., Liao X. W. (2017). Aldehyde dehydrogenase 1 (ALDH1) isoform expression and potential clinical implications in hepatocellular carcinoma. *PLoS ONE*.

[11] Shimamura M., Kurashige T., Mitsutake N., Nagayama Y. (2017). Aldehyde dehydrogenase activity plays no functional role in stem cell-like properties in anaplastic thyroid cancer cell lines. *Endocrine Journal*.

[12] Všianská P., Bezděková R., Kryukov F. (2017). Activity of aldehyde dehydrogenase in B-cell and plasma cell subsets of monoclonal gammopathy patients and healthy donors. *European Journal of Haematology*.

[13] Pal D., Kolluru V., Chandrasekaran B. (2017). Targeting aberrant expression of Notch-1 in ALDH+ cancer stem cells in breast cancer. *Molecular Carcinogenesis*.

[14] Bai S., Ingram P., Chen Y.-C. (2016). EGFL6 regulates the asymmetric division, maintenance, and metastasis of ALDH+ ovarian cancer cells. *Cancer Research*.

[15] Smith C., Gasparetto M., Humphries K., Pollyea D. A., Vasiliou V., Jordan C. T. (2014). Aldehyde dehydrogenases in acute myeloid leukemia. *Annals of the New York Academy of Sciences*.

[16] Gençer E. B., Yurdakul P., Dalva K., Beksaç M. (2017). Flow cytometric aldehyde dehydrogenase assay enables a fast and accurate human umbilical cord blood hematopoietic stem cell assessment. *Turkish Journal of Hematology*.

[17] Fallon P., Gentry T., Balber A. E. (2003). Mobilized peripheral blood SSCloALDHbr cells have the phenotypic and functional properties of primitive haematopoietic cells and their number correlates with engraftment following autologous transplantation. *British Journal of Haematology*.

[18] Roug A. S., Hokland L. B., Segel E. (2014). Unraveling stem cell and progenitor subsets in autologous grafts according to methods of mobilization: Implications for prediction of hematopoietic recovery. *Cytotherapy*.

[19] Gasparetto M., Sekulovic S., Brocker C. (2012). Aldehyde dehydrogenases are regulators of hematopoietic stem cell numbers and B-cell development. *Experimental Hematology*.

[20] Gasparetto M., Smith C. A. (2017). ALDHs in normal and malignant hematopoietic cells: Potential new avenues for treatment of AML and other blood cancers. *Chemico-Biological Interactions*.

[21] Muramoto G. G., Russell J. L., Safi R. (2010). Inhibition of aldehyde dehydrogenase expands hematopoietic stem cells with radioprotective capacity. *Stem Cells*.

[22] Gasparetto M., Sekulovic S., Zakaryan A. (2012). Varying levels of aldehyde dehydrogenase activity in adult murine marrow hematopoietic stem cells are associated with engraftment and cell cycle status. *Experimental Hematology*.

[23] Singh S., Brocker C., Koppaka V. (2013). Aldehyde dehydrogenases in cellular responses to oxidative/electrophilic stress. *Free radical biology and medicine*.

[24] Moreb J. S., Ucar-Bilyeu D. A., Khan A. (2017). Use of retinoic acid/aldehyde dehydrogenase pathway as potential targeted therapy against cancer stem cells. *Cancer Chemotherapy and Pharmacology*.

[25] Ghiaur G., Yegnasubramanian S., Perkins B., Gucwa J. L., Gerber J. M., Jones R. J. (2013). Regulation of human hematopoietic stem cell self-renewal by the microenvironment's control of retinoic acid signaling. *Proceedings of the National Acadamy of Sciences of the United States of America*.

[26] Chute J. P., Muramoto G. G., Whitesides J. (2006). Inhibition of aldehyde dehydrogenase and retinoid signaling induces the expansion of human hematopoietic stem cells. *Proceedings of the National Acadamy of Sciences of the United States of America*.

[27] Langevin F., Crossan G. P., Rosado I. V., Arends M. J., Patel K. J. (2011). Fancd2 counteracts the toxic effects of naturally produced aldehydes in mice. *Nature*.

[28] Rice K. L., Izon D. J., Ford J., Boodhoo A., Kees U. R., Greene W. K. (2008). Overexpression of stem cell associated ALDH1A1, a target of the leukemogenic transcription factor TLX1/HOX11, inhibits lymphopoiesis and promotes myelopoiesis in murine hematopoietic progenitors. *Leukemia Research*.

[29] Pearce D. J., Taussig D., Simpson C. (2005). Characterization of cells with a high aldehyde dehydrogenase activity from cord blood and acute myeloid leukemia samples. *Stem Cells*.

[30] Schubert M., Herbert N., Taubert I. (2011). Differential survival of AML subpopulations in NOD/SCID mice. *Experimental Hematology*.

[31] Ran D., Schubert M., Pietsch L. (2009). Aldehyde dehydrogenase activity among primary leukemia cells is associated with stem cell features and correlates with adverse clinical outcomes. *Experimental Hematology*.

[32] Cheung A. M. S., Wan T. S. K., Leung J. C. K. (2007). Aldehyde dehydrogenase activity in leukemic blasts defines a subgroup of acute myeloid leukemia with adverse prognosis and superior NOD/SCID engrafting potential. *Leukemia*.

[33] Gerber J. M., Smith B. D., Ngwang B. (2012). A clinically relevant population of leukemic CD34^+^CD38^−^cells in acute myeloid leukemia. *Blood*.

[34] Schuurhuis G. J., Meel M. H., Wouters F. (2013). Normal hematopoietic stem cells within the AML bone marrow have a distinct and higher ALDH activity level than co-existing leukemic stem cells. *PLoS ONE*.

[35] Hoang V. T., Buss E. C., Wang W. (2015). The rarity of ALDH+ cells is the key to separation of normal versus leukemia stem cells by ALDH activity in AML patients. *International Journal of Cancer*.

[36] Januchowski R., Wojtowicz K., Zabel M. (2013). The role of aldehyde dehydrogenase (ALDH) in cancer drug resistance. *Biomedicine & Pharmacotherapy*.

[37] Huang C.-P., Tsai M.-F., Chang T.-H. (2013). ALDH-positive lung cancer stem cells confer resistance to epidermal growth factor receptor tyrosine kinase inhibitors. *Cancer Letters*.

[38] Moreb J. S., Turner C., Sreerama L., Zucali J. R., Sladek N. E., Schweder M. (1995). Interleukin-1 and tumor necrosis factor alpha induce class 1 aldehyde dehydrogenase mRNA and protein in bone marrow cells. *Leukemia & Lymphoma*.

[39] Moreb J. S., Schweder M., Gray B., Zucali J., Zori R. (1998). In vitro selection for K562 cells with higher retrovirally mediated copy number of aldehyde dehydrogenase class-1 and higher resistance to 4-hydroperoxycyclophosphamide. *Human Gene Therapy*.

[40] Moreb J. S., Maccow C., Schweder M., Hecomovich J. (2000). Expression of antisense RNA to aldehyde dehydrogenase class-1 sensitizes tumor cells to 4-hydroperoxycyclophosphamide in vitro. *The Journal of Pharmacology and Experimental Therapeutics*.

[41] Purton L. E., Bernstein I. D., Collins S. J. (2000). All-trans retinoic acid enhances the long-term repopulating activity of cultured hematopoietic stem cells. *Blood*.

[42] Su M., Alonso S., Jones J. W. (2015). All-trans retinoic acid activity in acute myeloid leukemia: Role of cytochrome P450 enzyme expression by the microenvironment. *PLoS ONE*.

[43] Xu B., Wang S., Li R. (2017). Disulfiram/copper selectively eradicates AML leukemia stem cells in vitro and in vivo by simultaneous induction of ROS-JNK and inhibition of NF-kappaB and Nrf2. *Cell Death and Disease*.

[44] Conticello C., Martinetti D., Adamo L. (2012). Disulfiram, an old drug with new potential therapeutic uses for human hematological malignancies. *International Journal of Cancer*.

[45] Bista R., Lee D. W., Pepper O. B., Azorsa D. O., Arceci R. J., Aleem E. (2017). Disulfiram overcomes bortezomib and cytarabine resistance in Down-syndrome-associated acute myeloid leukemia cells. *Journal of Experimental & Clinical Cancer Research*.

[46] Venton G., Pérez-Alea M., Baier C. (2016). Aldehyde dehydrogenases inhibition eradicates leukemia stem cells while sparing normal progenitors. *Blood Cancer Journal*.

[47] Morgan C. A., Hurley T. D. (2015). Development of a high-throughput in vitro assay to identify selective inhibitors for human ALDH1A1. *Chemico-Biological Interactions*.

[48] Buchman C. D., Mahalingan K. K., Hurley T. D. (2015). Discovery of a series of aromatic lactones as ALDH1/2-directed inhibitors. *Chemico-Biological Interactions*.

